# Loop-Mediated Isothermal Amplification of Specific Endoglucanase Gene Sequence for Detection of the Bacterial Wilt Pathogen *Ralstonia solanacearum*

**DOI:** 10.1371/journal.pone.0096027

**Published:** 2014-04-24

**Authors:** Rok Lenarčič, Dany Morisset, Manca Pirc, Pablo Llop, Maja Ravnikar, Tanja Dreo

**Affiliations:** Department of Biotechnology and Systems Biology, National Institute of Biology, Ljubljana, Slovenia; Wageningen University and Research Centre, Netherlands; van Overbeek

## Abstract

The increased globalization of crops production and processing industries also promotes the side-effects of more rapid and efficient spread of plant pathogens. To prevent the associated economic losses, and particularly those related to bacterial diseases where their management relies on removal of the infected material from production, simple, easy-to-perform, rapid and cost-effective tests are needed. Loop-mediated isothermal amplification (LAMP) assays that target 16S rRNA, *fli*C and *egl* genes were compared and evaluated as on-site applications. The assay with the best performance was that targeted to the *egl* gene, which shows high analytical specificity for diverse strains of the betaproteobacterium *Ralstonia solanacearum*, including its non-European and non-race 3 biovar 2 strains. The additional melting curve analysis provides confirmation of the test results. According to our extensive assessment, the *egl* LAMP assay requires minimum sample preparation (a few minutes of boiling) for the identification of pure cultures and ooze from symptomatic material, and it can also be used in a high-throughput format in the laboratory. This provides sensitive and reliable detection of *R. solanacearum* strains of different phylotypes.

## Introduction

*Ralstonia solanacearum* is a Gram-negative soil-borne betaproteobacterium that is the causal agent of bacterial wilt disease, or potato brown-rot disease [1], [2]. *Ralstonia solanacearum* is listed as one of the most important plant pathogens [3], as its host range covers more than 450 plant species that belong to over 50 plant families [2], the majority of which are members of *Solanaceae* and *Musaceae* [4]. Among these, there are a number of economically important crops, including potato, tomato, eggplant, tobacco and pelargonium [5], [6], and therefore an effective diagnostic system for use during the production and importation of these plants and their products is needed [7].

The exceptional host range and evolution of *R. solanacearum* reflect the heterogeneous nature of this ‘species complex’ [8]. Based on phylogenetic grouping and deeply separated evolutionary lineages, *R. solanacearum* is divided into four phylotypes that reflect its geographic isolation and spatial distances: phylotypes I and II are composed of Asian and American strains, respectively, phylotype III members are of African origin, and phylotype IV isolates originate from Indonesia, Japan, and Australia, and include *R. syzigii* and blood disease bacteria (BDB). These phylotypes are further subdivided in sequevars that are based on differences in the sequences of a portion of the endoglucanase (*egl*) gene [9], [10]. Strains of *R. solanacearum* phylotype IIB, sequevar 1 (historically known as race 3 biovar 2) are of particular interest to Europe and North America, as they cause potato infections in temperate climates that can result in high economic losses [5]. Therefore, *R. solanacearum* is recognized as a quarantine pest in most countries, and by regional Plant Protection Organizations [11], [12].

The importance of infected propagative material of ornamental plants was shown by an outbreak of *R. solanacearum* in North America [7]. Due to the increased trade and/or movements of various host plants, in combination with changes in climatic conditions, there is an increased risk of the introduction and establishment of non-European *R. solanacearum* strains in both the environment and in greenhouse production facilities. This is exemplified by introduction of infected pelargonium and geranium cuttings through their importation [13]–[15] and the occurrence of other cold-tolerant strains [16]. In Europe, reports have demonstrated that *R. solanacearum* can survive in waterways at low temperatures in northern countries, and can cause infection of host plants after irrigation with contaminated water [17].

Several diagnostic methods are currently available to detect *R. solanacearum*. Serological techniques rely on the detection of bacterial components using antibodies [18], while molecular techniques are based on the detection of bacterial DNA [19], [20], or RNA of viable bacterial cells [21]. To control the spread of *R. solanacearum*, a rapid and more user-friendly test is needed that can be easily deployed on-site. Available on-site tests also include serological and molecular tests, such as real-time PCR with the portable SmartCycler (Cepheid, Sunnyvale, CA).

Our aim was to develop a loop-mediated isothermal amplification (LAMP) assay [22] that can be performed in a high-throughput format in the laboratory or can be used with a portable device based on real-time detection of *R. solanacearum* strains. Ideally, this would cover all four of the *R. solanacearum* phylotypes from its various host plants. The LAMP assays developed herein that target the 16S rRNA gene and the endoglucanase (*egl*) gene are compared to an optimized version of a previously published LAMP assay that targets the flagellar subunit *fli*C [23]. The best of these assays, the *egl* LAMP assay, is adapted for on-site performance with a portable device, and thus for real-time visualization. This is also compared to the real-time PCR assay developed by Weller and coworkers [19], which is routinely used for diagnostics in our laboratory.

## Methods

### Sample preparation

#### Bacterial isolates

The LAMP assays were evaluated on 114 bacterial strains that included 88 *R. solanacearum* ‘species complex’ strains that represent all four of the phylotypes: phylotypes I (20), IIA (17), IIB (24), III (15), and IV (8). Four strains with undetermined phylotypes were also included. In addition, the LAMP assays were evaluated on 13 non-target strains, which included potentially cross-reacting reference strains (as listed in European Union Council Directive 2000/29/EC) [24], plus 13 other pathogens from selected *R. solanacearum* hosts (for full listing and additional information, see Table S4 in File S1).

The bacterial strains were grown on YPGA agar medium (0,5% yeast extract, 0,5% peptone, 1% glucose and 1,2% agar) and incubated at 28 °C for two days. A single colony of each was then suspended in 10 mM phosphate-buffered saline (PBS; pH 7.2) to a final concentration of 10^8^ cells/mL, which was standardized by turbidity measurements (DEN-1B McFarland Densitometer, Biosan). The bacterial suspensions were incubated at 95 °C for 30 min in a thermal block, to lyse the cells and release their DNA, prior to serial 10-fold dilutions in distilled water (10^8^-10 cells/mL). The DNA dilutions were stored at −20 °C until analysis.

#### Preparation of plant material

Plants and plant parts were inoculated with *R. solanacearum* to produce the necessary plant material that mimicked naturally infected symptomatic plants.

Tomato plants at the third true leaf stage were inoculated with pure cultures of *R. solanacearum* (NCPPB 1453 or NCPPB 4156) using a sterile needle. The plants were incubated at 90% relative humidity under a regime of 16 h light (3000 lux) at 26 °C and 8 h darkness at 23 °C. When the plants showed wilting symptoms (2–4 days after inoculation), the bacterial ooze at the site of inoculation was collected using a sterile plastic inoculation loop, and suspended in 100 µL sterile water. For additional confirmation, after a week of incubation, the plant stems above the inoculation point were cut into pieces and tested using the LAMP assay. Artificially infected potato tubers were: (i) stabbed at the stolon end using an inoculation needle dipped in an *R. solanacearum* colony, and then incubated at 28 °C and 70% humidity for 2–3 weeks; or (ii) collected from artificially infected plants (two months old potato plants of Fontane variety were infected by irrigation with 10^6^ cells/mL of *R. solanacearum* strain LMG 9576 and tubers were then collected after three months). Infected tubers were cut into halves and the bacterial ooze was collected with a sterile plastic inoculation loop and suspended in 100 µL sterile water.

The ooze (2.5 µL) of both, artificially inoculated tomato plants and potato tubers was tested in the LAMP reactions as described below.

Infected and healthy potato tuber extracts used for testing latent *R. solanacearum* infection were prepared using official EC procedure [24], by cutting out small cores of vascular tissue from the stolon end of each of a sample of 200 tubers, and comminuting these in 30 mL 50 mM phosphate buffer (pH 7.0), with an overnight incubation (shaking; maximum, 16 h). The supernatant was then decanted into a 50-mL centrifuge tube and centrifuged at 7000× *g* for 15 min at 6 °C. The pellet was resuspended in 1 mL 10 mM phosphate buffer (pH 7.2), and the DNA was isolated as described below. The DNA (2.5 µL) isolated from the extract was then tested in the LAMP reactions.

Tuber extracts of potato cultivars Désirée and Bella Rosa were spiked with a 10-fold dilution series of *R. solanacearum* NCPPB 4156 (phylotype IIB), which ranged from 10^6^ to 1 cell/mL, followed by DNA extraction. The isolated DNA from the spiked potato tuber extracts was then used for determination of analytical sensitivity.

Extracts of healthy plants of other hosts (tomato (4), eggplant (3), pelargonium (6) and *Solanum dulcamara* (6)) were prepared by cutting approximately 0.1 g of the plant stems into pieces and comminuting them in 4 mL 10 mM PBS (pH 7.2). The liquid part was removed and 100 µL was used for DNA extraction.

#### DNA extraction

For conventional DNA extraction, 100 µL of the extracts from each potato sample or from healthy plants of tomato, eggplant, pellargonium and *S. dulcamara* were included, using magnetic-bead-based QuickPick SML Plant DNA kits (Bio-Nobile) with the KingFisher mL system (Thermo Labsystem), according to Pirc *et al*., 2009 [25], with a minor modification (440 µL lysate used for purification).

The resuspended ooze from the artificially infected tomato plants and potato tubers were incubated at 95 °C in a thermal block for 5 min and 2 min, respectively, to lyse the cells and to release the DNA. Then 2.5 µL of this solution was tested in the LAMP reaction.

Furthermore, different incubation times of the homogenate at 95 °C were tested with pure bacterial cultures: 2, 5, 10, 15, 20 and 30 min, and these were compared with the time needed to obtain positive results from symptomatic test material.

### The LAMP assay

The performance of the *egl* LAMP assay is described in the following section. Readers are referred to Supplementary Materials for descriptions of the 16S rRNA LAMP assay (Table S1 in File S1) and the optimized *fli*C LAMP assay (Table S2 and Table S3 in File S1).

#### Design of the LAMP primers

All of the available sequences of the *R. solanacearum* endoglucanase genes were collected at the time of analysis, from the NCBI database, available at http://www.ncbi.nlm.nih.gov/genbank/. These were aligned using the muscle alignment algorithm and the Molecular Evolutionary Genetics Analysis v5 (Mega 5) software [26]. The consensus (98% conservation) of the most conserved part of the alignment, identical to sequence GenBank accession number DQ657595, was then used for the design of the LAMP primers, using the LAMP Designer software (Premier Biosoft, Palo Alto, CA). The standard BLAST algorithm (standard nucleotide BLAST; http://blast.ncbi.nlm.nih.gov/Blast.cgi) was used for each primer set with the default settings, to check for specificity against the whole database and for cross-reactivity with other nontarget sequences, including bacterial and plant sequences. Using this strategy, three primer sets that target the endoglucanase (*egl*) gene were designed, all of which satisfied the required parameters described by Notomi *et al*. [22]. After preliminary sensitivity and specificity assessments on a set of 14 isolates that represented all of the *R. solanacearum* phylotypes (data not shown), one primer set was chosen for further validation (Table 1).

**Table 1:** Primers used in the *egl* LAMP assay.

Primer	5′-3′ primer sequence	Final primer conc. (μM)	Primer position*
F3_RS_egl	GAGCAACTACATCTATCCGTC	0.2	330–350
B3_RS_egl	CATCAGCCCGAAGATGAC	0.2	637–654
FIP_RS_egl	ACAGCTCGTTCGCGTCGACGACAGCGCGACCTACTA	2	354–371, 446–463
BIP_RS_egl	GGTTCGTCAACGCCGTGAGATCACGTTGCCGTAGTAG	2	476–493, 540–558
FLoop_RS_egl	GTTCATGCCCTTGTTCTTG	2	372–390
Bloop_RS_egl	GCTCGATCCGCACAACTA	2	516–533

*Position of the primer on *R. solanacearum* strain GMI100 endoglucanase precursor (*egl*) gene (GenBank accession number DQ657595) sequence.

#### LAMP reactions

The LAMP reactions were performed in single tubes in 25 µL total reaction volume, containing 2.5 µL sample (boiled bacterial suspension or DNA isolated from plant extract), 12.5 µL Isothermal Master Mix (Optigene Ltd., Horsham, UK), and the primer mix that consisted of all six primers, at the final concentrations as indicated in Table 1. The *egl* LAMP reactions were run at 60 °C for 30 min. All of the reactions for evaluation of the test were performed in a SmartCycler instrument (Cepheid, Sunnyvale, CA), using a negative control (distilled water) and a positive control (*R. solanacearum* strain GBBC 1172) for every run. The reactions for the on-site performance of the test were performed using the Genie II equipment (Optigene Ltd., Horsham, UK). Fluorescence was followed in real time in the FAM fluorescence channel.

#### Positivity criteria and running-time determination

The criteria for positive signals were based on two parameters: t_p_ (time of positivity), and T_m_ (melting temperature). The t_p_ is the amplification time expressed in minutes at which the fluorescent second derivative reaches its peak above the baseline value. The T_m_ is the temperature at which the amplification products melt into two single-stranded DNA molecules, and this is expressed in °C.

The running time for the *egl* LAMP assay was determined based on the sensitivity test results from the *R. solanacearum* strain that showed the latest time of positivity (t_p_) at the limit of detection for each assay. Based on these data, the reaction running time was optimal and sufficient to detect all of the *R. solanacearum*-positive samples (Table S4 in File S1). This approach supports optimum assay performance by allowing target detection in samples where the bacterial concentration is at the limit of detection, while preventing the appearance of eventual signals from nonspecific amplification, which is however distinguishable based on their melting curves.

### Real time PCR

All of the samples tested with the *egl* LAMP assay were also analyzed by real-time PCR using the primers RS-I-F and RS-II-R that flank the 16S rRNA region, as suggested for broad-range detection of all *R. solanacearum* biovars [19]. The real-time PCR reactions were performed in triplicate on an ABI PRISM 7900 HT Sequence Detection system (Applied Biosystems), using the following universal cycling conditions: 2 min at 50 °C, 10 min at 95 °C, followed by 45 cycles of 15 s at 95 °C, and 1 min at 60 °C, using standard mode. The reaction volumes of 10 µL contained, as final concentrations: 900 nM primers (Eurofins MWG Operon), 200 nM probe (Eurofins MWG Operon), 1× TaqMan Universal PCR Master Mix (Applied Biosystems), and 2 µL sample DNA. The real-time PCR results are given in Cq (quantification cycle): the real-time PCR cycle at which the fluorescence exceeded the threshold value [27].

### Confirmational methods

For the determination of health status of the potato tuber extracts, the diagnostics followed EC Directive 2000/29/EC [24]: immunofluorescence microscopy was used as a screening test with the IACR-PS-278 anti-*R. solanacearum* (biovar 2) polyclonal rabbit primary antibody (Rothamstead Research) [28], with a goat anti-rabbit FITC-labeled secondary antibody (Sigma F6005). Tuber extracts with positive results in the immunofluorescence were further analysed using PCR (primers Rs-1-F, Rs-1-R; [29]), isolation on semi-selective SMSA medium, immunofluorescence, and PCR on pure cultures, and pathogenicity testing in tomato plants cv. Moneymaker. In addition, negative tuber extracts in immunofluorescence were further tested with real-time PCR that targeted the 16S rRNA gene using a broad range *R. solanacearum* probe [19].

### Analytical specificity assays

Analytical specificity was determined on boiled suspensions of 10^8^ cells/mL (*egl* LAMP assay) or 10^6^ cells/mL (real-time PCR), standardized by turbidity measurements (DEN-1B McFarland Densitometer, Biosan). Comparisons with real-time PCR were carried out as described above.

The cross-reactivity of the LAMP assays with the potato plant tissue and its microflora was assessed by testing DNA isolated from potato tuber extracts that had previously tested negative using other methods (immunofluorescence and/or real-time PCR). The following cultivars were included: Adora (2), Agria (1), Aladin (1), Anuschka (1), Arrow (2), Bella Rosa (1), Bistra (1), Carlingford (3), Carrera (1), Cherie (1), Desiree (2), Fiana (1), Frisia (2), Jelly (3), Marabel (7), Mirna (1), Pšata (1), Sante (3), Silvana (1), Sora (3), Soraja (1), two samples of an unknown cultivar, and two samples of a new hybrid (Table S5 in File S1). In addition, DNA isolated from extracts of the following healthy plant tissues were tested: tomato (4), eggplant (3), pelargonium (6) and *S. dulcamara* (6). The health status of these plant extracts was confirmed by real-time PCR for broad range detection of *R. solanacearum*, and by immunofluorescence microscopy, as described above.

### Analytical sensitivity assays

Analytical sensitivity was evaluated on pure bacterial cultures diluted in distilled water. A 10^8^ cells/mL suspension of a representative strain for each phylotype was prepared. This suspension was then used for the preparation of 10-fold dilution series on which the analytical sensitivity was evaluated in triplicate with the LAMP assay, and compared to real-time PCR, as described above.

In addition, the analytical sensitivity of the LAMP assay in the potato tuber samples was determined on spiked potato extracts prepared as described above. The *R. solanacearum* concentrations in tuber extracts ranged from 10^6^ to 1 cell/mL. DNA was extracted from 100 µL samples and analyzed with real-time PCR and LAMP assays, as described above.

For the purpose of this study, the limit of detection was determined for each technique, which was defined as the lowest dilution where two out of three and one out of three reactions were positive in LAMP and real-time PCR, respectively.

## Results

### Performance of the LAMP assays

Three LAMP assays are described and compared in this study: the in-house developed LAMP assays that target the 16S rRNA and endoglucanase (*egl*) genes, and the previously developed LAMP assay that targets *fli*C, which was optimised for real-time performance and made more rapid by the addition of another loop primer. A summarized evaluation scheme and the criteria for choosing the LAMP assays with better performances are presented in the Supplementary Materials (Figure S1).

**Figure 1:**
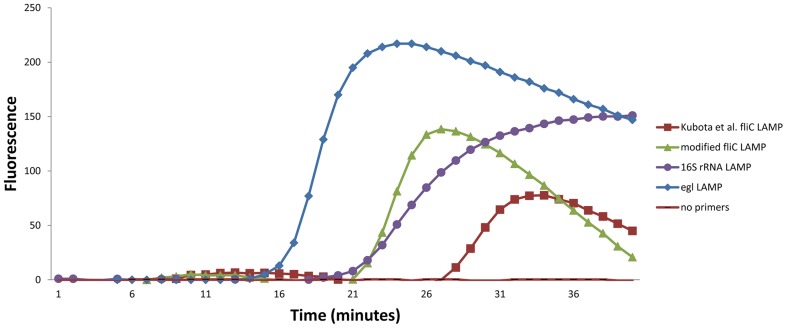
Comparison of the amplification speeds of the different LAMP assays. When tested on *R. solanacearum* strain NCPPB 3997 (10^8^ cells/mL), the *egl* LAMP assay (blue) was the fastest assay, compared with the modified *fli*C (green) and 16S rRNA (violet) assays.

All three LAMP assays successfully amplified the DNA of the NCPPB 3997 *R. solanacearum* isolate, as shown on Figure 1. However, due to cross-reactivity with DNA from healthy potato extracts, the 16S rRNA LAMP assay was not suitable for diagnostic testing, and this was therefore removed from further evaluation. The optimized *fli*C LAMP assay showed a better performance than the 16S rRNA LAMP assay in terms of specificity, sensitivity, and speed, but it did not detect several economically important *R. solanacearum* strains, and this was therefore not selected for on-site application. The *egl* LAMP assay was the fastest of these tested LAMP assays, and the time to result when tested at 10^8^ cells/mL ranged from 12 min to 20 min for the majority of the isolates (Table S4 in File S1). Based on the speed and the above-mentioned criteria, the running time for the *egl* LAMP assay was set to 30 min.

### Analytical specificity assays

The analytical specificity was tested on pure bacterial cultures. The *egl* LAMP assay detected all of the isolates that belonged to the phylotypes I, IIA, IIB, and III, and four out of eight strains currently classified into phylotype IV. The phylotype IV strains that were not detected included one *Ralstonia syzygii* strain (CFBP6447), both of the BDB strains tested, and *R. solanacearum* strain RUN14/ACH732, which is genetically distinct from the other phylotype IV strains [30].

The analytical specificity of the *egl* LAMP assay was compared to that of the real-time PCR assay that was used to detect a broad range of *R. solanacearum* isolates [19]. This latter assay detected all of the tested *R. solanacearum* strains, as well as *R. syzygii* and the BDB strains, with the notable exception of the same phylotype IV *R. solanacearum* RUN14 strain that was not detected by the *egl* LAMP assay (Table S4 in File S1). Real-time PCR, but not *egl* LAMP, also detected the non-target *Ralstonia mannitolilytica* strain (CFBF6737).

To examine potential cross-reactivity with, e.g., soil microflora or plant DNA, the DNA samples isolated from healthy potato extracts (as confirmed by immunofluorescence with the sensitivity of 10^4^ cells/mL for biovar 2 *R. solanacearum*) were tested with the *egl* LAMP assay and by real-time PCR. The *egl* LAMP assay showed positive signals of amplification for four of the tested healthy potato samples (Table S5 in File S1). However, the melting curve analysis of the positive signals showed significantly different melting temperatures (difference of 1 °C or more) compared to the melting curve defined for the true positive signals obtained by *egl* LAMP. Based on the defined criteria of positivity (Table S4 and Table S2 in File S1), these data were classified as negative, and did not cause problems in the interpretation of the results.

In contrast, four samples of potato tuber extracts that were negative using the official detection methods which are immunofluorescence microscopy and PCR [29] gave false-positive results in real-time PCR. Cross-contamination was excluded using appropriate negative and positive controls for DNA extraction and amplification.

In addition, to test the on-site applicability, bacterial ooze from seven symptomatic tubers was tested. All of these samples showed positive signals, with t_p_ of 18.8 min (±1.88 min) and T_m_ of 93.2 °C (±0.07 °C), both of which are characteristic of the *R. solanacearum* endoglucanase amplicon.

### Melting curve analysis

**Figure 2:**
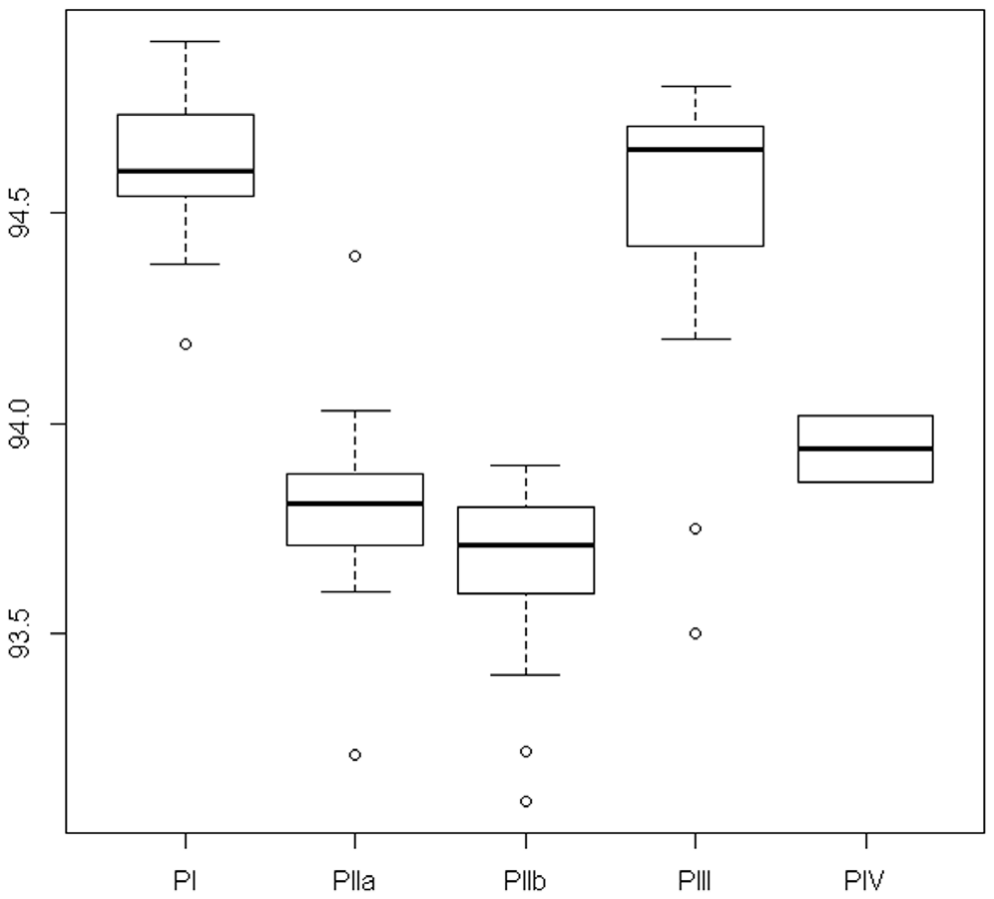
Melting temperature ranges of the LAMP products obtained on *R. solanacearum* strains using the *egl* LAMP assay. PI, PIIA, PIIB, PIII, PIV: phylotypes I, IIA, IIB, III and IV, respectively. The melting temperatures were measured on a SmartCycler apparatus (Cepheid, Sunnyvale, CA).

Although the *egl* gene is present in all of the *R. solanacearum* strains, the geographical separation and consequent separation of the evolution of the *R. solanacearum* species resulted in specific nucleotide substitutions in the *egl* gene sequence; this allows the differentiation of the phylotypes. From the results of our specificity assessment of the *egl* LAMP assay, *R. solanacearum* can be divided into two groups based on the melting temperatures observed for the LAMP products (Figure 2). The T_m_ of LAMP products obtained in the amplification of strains that belong to phylotypes I (Asia) and III (Africa) were 94.6 °C (±0.2 °C) and 94.5 °C (±0.4 °C), respectively, whereas T_m_ of the LAMP products of the strains of phylotypes IIA and IIB (America) were 93.8 °C (±0.2 °C) and 93.7 °C (±0.2 °C), respectively. This is in agreement with the reported phylogenetic studies: phylotypes IIA and IIB are closely related compared to phylotypes I and III [30]. Phylotype IV is a very heterogenous group, and it was tested with a limited number of strains (8), and the T_m_ fell into the same range: 94.1 °C (±0.3 °C). Overall, the range of T_m_ found with the *egl* LAMP assay for *R. solanacearum* was between 93.1 °C and 94.9 °C, thus defining a criterion for LAMP signal acceptability (true positive).

### Analytical sensitivity assays

The sensitivity was first evaluated on pure bacterial cultures diluted in distilled water, using a representative strain for each phylotype (Table 2). The *egl* real-time LAMP assays showed a sensitivity limit of 10^4^ cells/mL (25 cells per LAMP reaction) when tested on strains belonging to phylotypes I and III, and a sensitivity limit of 10^5^–10^6^ cells/mL for strains from phylotypes IIA, IIB and IV (Table 2).

**Table 2:** Analytical sensitivity of the *egl* LAMP assay.

Concentration (cells/mL)	GBBC 1172 (Phylotype I)	RUN 30 (Phylotype IIA)	GBBC 729 (Phylotype IIB)	LMG 2296 (Phylotype III)	RUN 71 (Phylotype IV)
	t_p_ (min)	t_p_ (min)	t_p_ (min)	t_p_ (min)	t_p_ (min)
10^8^	12.7 ±0.38	15.8 ±1.59	12.9 ±1.07	12.2 ±0.06	19.4 ±1.67
10^7^	14.2 ±1.81	16.5 ±2.73	14.7 ±1.76	13.8 ±1.56	18.8 ±1.90
10^6^	17.0 ±3.03	18.1 ±3.97	16.3 ±1.86	15.7 ±2.03	19.3 ±2.44
10^5^	18.1 ±3.13	23.3^a^	17.7 ±2.41^b^	17.4 ±2.02	21.3^a^
10^4^	20.8 ±3.99	26.3^a^	21.3^a^	20.5 ±2.28	-
10^3^	-	-	-	-	-
10^2^	-	-	-	-	-
T_m_	94.1 ±0.10	93.5 ±0.27	93.2 ±0.08	94.1 ±0.14	93.8 ±0.25

Data are means (±SD), calculated from three independent runs.

“-”: negative result (absence of signal).

a , detected once out of three replicates.

b , detected twice out of three replicates.

Data obtained on different *R. solanacearum* phylotypes samples, from boiled bacterial suspensions.

In addition, the sensitivity of the *egl* LAMP assay was evaluated in spiked plant extracts, to simulate real plant samples and to confirm that the level of sensitivity is not influenced by substances co-extracted with the DNA from the plant tissues. To test a worst case scenario, a strain with poor LAMP performance (NCPPB 4156) was tested. The sensitivity for the spiked potato extract was estimated to be 10^5^ CFU/mL.

### On-site application

To offer an *egl* LAMP assay that is suitable for application outside the laboratory, a quick and simple extraction protocol (boiling of samples) of symptomatic potato tubers was combined with LAMP, using the portable Genie II equipment. In general, the longer boiling times resulted in shorter times to the signal of the LAMP reactions (Table S7 in File S1). A boiling time of 2 min at 95 °C was repeatedly successful for the confirmation of tuber infections (six tubers tested). Positive signals using the *egl* LAMP assay had a t_p_ of 18.4 ±1.07 min and a T_m_ of 92.1 ±0.11 °C, which were characteristic for *R. solanacearum* when tested on the Genie II apparatus. Testing symptomatic tomato plants (infected with *R. solanacearum* strains NCPPB 4153 and NCPPB 4156) showed comparable results (data not shown). Taken together, these data gave good indications that the test can be easily performed on-site, with reliable results obtained.

## Discussion

Our investigation focused on the development of a molecular assay for rapid testing of symptomatic material. The availability of portable equipment for on-site performance that allows LAMP amplification to be followed in real-time was another reason for this study.

Of the three assays compared here, the LAMP assay that targets the *egl* gene appears to be optimal for testing symptomatic material, in terms of specificity, sensitivity and time needed to complete the analysis (Tables 2, 3 and Tables S4, S5, S8 in File S1).

**Table 3:** Performance characteristics of the LAMP assays compared with real-time PCR.

Assay characteristic	16S rRNA LAMP	Modified *fli*C LAMP	*egl* LAMP	Real-time PCR
Target gene	16S rRNA	Flagellar subunit	Endoglucanase	16S rRNA
Running temperature (°C)	65	65	60	Cycling
Running time* (min)	60	40	30	90
Analytical sensitivity in water^a^ (cells/mL)	ND**	10^5^–10^8^	10^4^–10^6^	10^2^–10^4^
Analytical sensitivity in tuber extract^b^ (CFU/mL)	10^3^–10^4^	10^5^	10^5^	10^3^–10^2^
10^6^	31.0 ±0.12	16.5 ±0.70	19.8 ±0.41	24.2 ±0.03
10^5^	34.1 ±1.52	22.5 ±5.00	20.2 ±5.01	27.7 ±0.08
10^4^	43.7 ±3.91	/	/	31.5 ±0.40
10^3^	49.6	/	/	39.8 ±1.38
10^2^	/	/	/	/
10^1^	/	/	/	/
0	/	/	/	/

*Running time: time needed to complete the test with reliable detection of all samples above the limit of detection.

a Analytical sensitivity was tested on a pure culture suspension of a single strain representing each phylotype (see Table 2). Analytical sensitivity differs between phylotypes. More detailed results are presented in Table 2 (*egl* LAMP assay) and Table S3 in File S1 (modified *fli*C LAMP assay) and Table S6 in File S1 (real time PCR).

b Analytical sensitivity tested on DNA isolated from spiked tuber extracts (with DNA of strain NCPPB 4156 that belongs to phylotype IIB) was assessed on two serial dilutions.

**ND: Non-determined. Analytical sensitivity in spiked plant extract was not tested with the 16S rRNA LAMP assay because of cross reactivity observed; therefore validation was not completed for this assay.

This *egl* LAMP assay covers all of the tested *R. solanacearum* strains from phylotypes I, II and III, and most of the tested *R. solanacearum* strains from phylotype IV. Most importantly, all of the strains within the four phylotypes that have been reported to be found in potato were correctly detected. In addition, the *egl* LAMP assay confirmed its excellent specificity when tested on carefully chosen nontarget samples.

The proposed *egl* LAMP assay presents further advantages in terms of detection. It detects all of the bacteria from clonal phylotype IIB and the genetically more diverse phylotype IIA [30]. Therefore, the *egl* LAMP assay is a relatively sustainable detection test for the phylotype IIB strains, that includes previously designated race 3 biovar 2 isolates, which are classified as a quarantine isolates in European Union and Canada [31]. Moreover, the *egl* LAMP assay is not restrained to the strains that are currently causing economic losses, but can also detect all of the strains from phylotype I, the phylotype that has the biggest evolutionary potential and also the largest host range [30]. As only three *R. solanacearum* phylotype IV strains were tested (including the genetically distinct RUN14/ACH732 strain), it is difficult to forecast how efficient the *egl*-specific LAMP assay will be for the detection of strains belonging to this phylotype. However, a recent genomic comparison revealed that strains classified into phylotype IV *R. solanacearum*, *R syzygii* and the BDB strains show high levels of evolutionary homology and might actually be considered as species distinct from *R. solanacearum* [32].

In terms of the time of the analysis, the *egl* LAMP assay is also the most rapid of these three assays, as only a 30-min reaction is necessary to complete the detection of all of the *R. solanacearum* strains.

Although the sensitivity of the *egl* LAMP assay (10^4^–10^6^ cells/mL) is lower than that of the real-time PCR (10^2^–10^4^ cells/mL) [19], its level of sensitivity is suitable for reliable confirmation of the presence of *R. solanacearum* in symptomatic potato samples. Moreover, data obtained in our laboratory show that latent infections of potato tubers usually contain bacterial concentrations of 10^6^ CFU/mL or higher (Table S5 in File S1). This suggests that the sensitivity of the *egl* LAMP assay may be sufficient for testing of latent infections.

Given the good specificity of this *egl* LAMP assay when tested on pure cultures, and that there were no false positives when compared to real-time PCR, we propose this assay to be validated for the confirmation of results that have been obtained using other diagnostic methods, such as immunofluorescence, selective isolation, and PCR. In addition to melting temperatures, the sequencing of the LAMP products provides further information on the phylogenetic identity of the isolates. Based on partial cds of the endoglucanase (*egl*) gene of a subset of *R. solanacearum* strains, several nucleotides provide good indications of the phylogenetic position of isolates; relative to the *R. solanacearum* strain CFBP 4599 *egl* sequence (GeneBank accession number GU294973), nucleotides T (463), A (519) and T (554) are indicative of phylotype II, nucleotides C (465) and C (500) of phylotypes III and I, and nucleotides A (479), T (484) and G (504) of phylotype IV, BDB and *R. syzygii* (Figure S2).

The real-time detection of LAMP products described here based on increased fluorescence signals is much simpler than turbidity/colorimetric detection, where the testing time can be up to 3 h [23], compared to only 40 min (including hands-on work) for the real-time *egl* LAMP assay. Moreover, the use of real-time detection allows the end user to stop the reaction once clear positive signals appear, and thus to proceed to the next step: melting curve analysis. The melting curve analysis is another benefit of real-time-based LAMP detection, as it offers additional confirmation of amplification results. The use of an intercalating dye in combination with melting curve analysis, or alternatively, of probe-based LAMP [33], represents a reliable way to exclude any possible miss-priming that has been reported to occur in some LAMP assays [34], and which cannot be distinguished from true positives when the LAMP product is detected by turbidimetry [33].

As well as providing a simple detection system that can be deployed on-site, a short and efficient nucleic acid extraction step is crucial for on-site applicable assays. Sufficient amounts of nucleic acid should be extracted in a short time, which was reached by incubation of the samples at 95 °C. Although no significant linear dependence was noted, the longer incubation times were generally seen to speed up the LAMP reactions that follow the pre-incubation, while the shorter times generally delayed the LAMP reactions. This might be because the longer the incubation time is, the better DNA denaturation is, resulting in more accessible DNA. However,a 2-min-long incubation was sufficient for the testing of symptomatic potato tubers (Table S7 in File S1).

The above-described characteristics make the *egl* LAMP assay suitable for on-site testing of symptomatic potato material at ports or other entry points, to discriminate between brown rot and other diseases. The test can also be performed at the place of production of potato, or of other plants that are potential hosts of *R. solanacearum*, because the test is confirmed to detect strains isolated from various host plants. This makes the test also relevant for application to locations where isolates other than phylotype IIB can be expected; e.g., in greenhouses, or in wastewaters from greenhouses.

## Conclusions

The herein described endoglucanase (*egl*)-specific real-time LAMP assay allows specific detection of *R. solanacearum* strains belonging to all four phylotypes. The assay is performed with a portable instrument that is fully suitable for on-site extraction and testing at the port of entry of plant material. It allows simple manipulation, fast analysis, and easy interpretation of the results. Its practicability has already been shown on-site, combined with boiling as a simple DNA extraction step. In addition, the assay can easily be implemented in laboratories in a high-throughput format, using their own real-time PCR instruments. Given its sensitivity level, this generic *R. solanacearum* detection assay is proposed for use as a detection tool for the presence of this bacterium in symptomatic plant material, although it can also be used as a rapid confirmation tool for pure bacterial colony identification.

## Supporting Information

Figure S1 **Scheme of evaluation of different LAMP assays and the decision scheme for choosing LAMP with best performance.**
(DOC)

Figure S2 **Alignment of partial cds of the endoglucanase (** ***egl*** **) gene of a subset of** ***R. solanacearum*** **strains.**
(JPG)

File S1 Supporting information that includes: Table S1: Primers used in the 16S rRNA LAMP assay. Table S2: Primers used in the modified *fli*C LAMP assay. Table S3: Analytical sensitivity of the modified *fli*C LAMP assay in different *R. solanacearum* phylotypes. Table S4: Bacterial strains tested with LAMP targeting 16S rRNA, *fli*C and *egl* compared to real-time PCR. Table S5: Evaluation of LAMP performance on potato extracts (diagnostic samples). Table S6: Analytical sensitivity of the real-time PCR for the different *R. solanacearum* phylotypes. Table S7: Incubation times at 95 °C before the assays, and the following time to positivity and time taken for the *egl* LAMP assays with infected potato tuber. Table S8: Summary of the *egl* LAMP validation.
(DOCX)
