# Supplementary material for: Loop-Mediated Isothermal Amplification of Specific Endoglucanase Gene Sequence for Detection of the Bacterial Wilt Pathogen Ralstonia solanacearum
Source: PLoS One. 2014 Apr 24;9(4):e96027. doi: 10.1371/journal.pone.0096027 (PMC3999105; doi:10.1371/journal.pone.0096027)

Figure S1: Scheme of evaluation of different LAMP assays and the decision scheme for choosing LAMP with best performance.


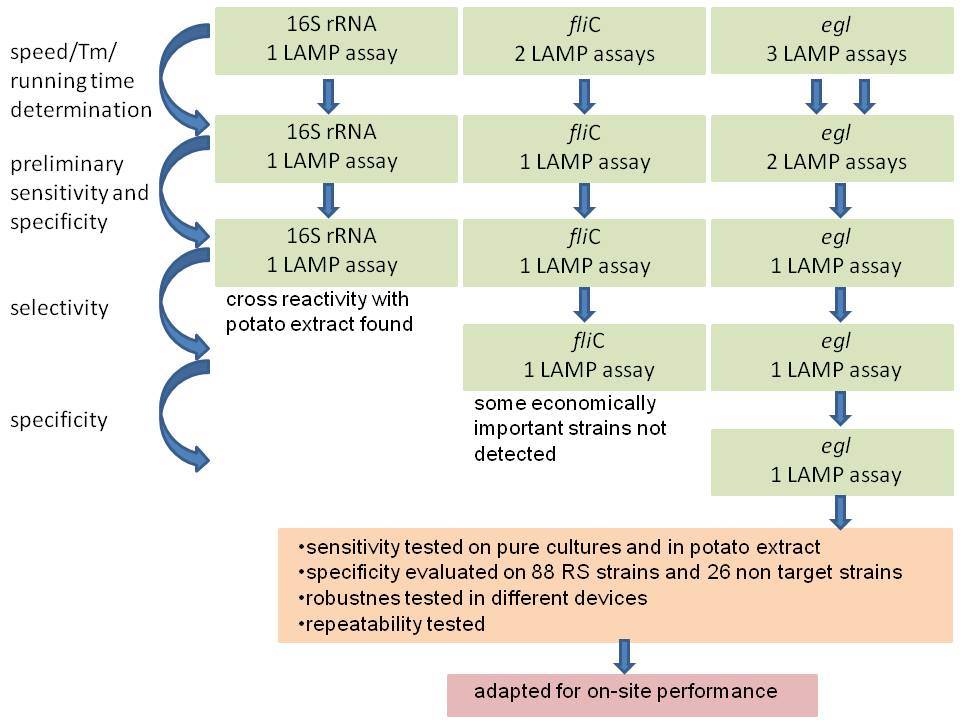

Supplement: Figure S1 — Scheme of evaluation of different LAMP assays and the decision scheme for choosing LAMP with best performance. (DOC) [file pone.0096027.s001.doc]
