# Supplementary material for: Loop-Mediated Isothermal Amplification of Specific Endoglucanase Gene Sequence for Detection of the Bacterial Wilt Pathogen Ralstonia solanacearum
Source: PLoS One. 2014 Apr 24;9(4):e96027. doi: 10.1371/journal.pone.0096027 (PMC3999105; doi:10.1371/journal.pone.0096027)
Supplement: File S1 — Supporting information that includes: Table S1: Primers used in the 16S rRNA LAMP assay. Table S2: Primers used in the modified fliC LAMP assay. Table S3: Analytical sensitivity of the modified fliC LAMP assay in different R. solanacearum phylotypes. Table S4: Bacterial strains tested with LAMP targeting 16S rRNA, fliC and egl compared to real-time PCR. Table S5: Evaluation of LAMP performance on potato extracts (diagnostic samples). Table S6: Analytical sensitivity of the real-time PCR for the different R. solanacearum phylotypes. Table S7: Incubation times at 95 °C before the assays, and the following time to positivity and time taken for the egl LAMP assays with infected potato tuber. Table S8: Summary of the egl LAMP validation. (DOCX) [file pone.0096027.s003.docx]

# Supplementary information

## 1. 16S rRNA LAMP

### *1.1 LAMP assay development*

All of the available sequences of the *R. solanacearum* 16S rRNA gene were collected from the NCBI database at the time of analysis, available at <http://www.ncbi.nlm.nih.gov/genbank/>. These sequences were aligned using the muscle alignment algorithm and the Molecular Evolutionary Genetics Analysis v5 (MEGA 5) software [26]. The consensus (98% conservation) of the most conserved part of the target sequence was then used for the design of the LAMP primers. One primer set targeting 16S rRNA was designed that satisfied the required parameters described by Notomi *et al*. [22] using the LAMP Designer software (Premier Biosoft, Palo Alto, CA). The standard BLAST algorithm (standard nucleotide BLAST available at <http://blast.ncbi.nlm.nih.gov/Blast.cgi>) was used for each primer set, with the default settings, to check for specificity against the whole database and for cross-reactivity with other non-target sequences, including bacterial and plant sequences.

### *1.2 LAMP reactions*

LAMP reactions were performed as described in the Methods using primers that are listed in Table S1.

Table S1. Primers used in the 16S rRNA LAMP assay.

| **Primer** | **5’-3’ primer sequence** | **Final primer conc.**  **(**µM**)** |
| --- | --- | --- |
| F3_RS_16S rRNA | TGATCCTGGCTCAGATTGA | 0.2 |
| B3_RS_16S rRNA | CACTTTCACCCTCAGGTC | 0.2 |
| FIP_RS_16S rRNA | ATTACTCACCCGTTCGCCACTGCCTTACACATGCAAGTC | 1 |
| BIP_RS_16S rRNA | ACATCGGAACGTGCCCTGGTATGCGGTATTAGCTAGTCTT | 1 |
| FLoop_RS_16S rRNA | CGGCAGGTAGCAAGCTAC | 1 |
| Bloop_RS_16S rRNA | AGTGGGGGATAACTAGTCGA | 1 |

### *1.3 Analytical specificity*

The analytical specificity was determined on boiled suspensions of 10^6^ cells/mL, as determined by turbidity measurements (DEN-1B McFarland Densitometer, Biosan). Comparisons with real-time PCR were carried out as described in the Methods.

### *1.4 Results*

Based on the described criteria for the speed and running time, the running time was set to 60 min for the 16S rRNA LAMP assay.

The analytical specificity of the 16S rRNA LAMP assay was tested on boiled bacterial suspensions at 10^6^ cells/mL, as listed in Table S4. All of the strains belonging to the *R. solanacearum* species complex were detected. No signals were obtained for possible cross-reacting bacterial strains listed in the European Union Council Directive 2000/29/EC [24] or on other pathogens that might be present in potato tubers (Supplementary Table S4). The range for the melting temperatures of the 16S rRNA LAMP amplicons measured for the *R. solanacearum* strains was between 88.4 °C and 90.2 °C.

However, using the 16S rRNA LAMP assay, a significant proportion of the healthy potato tuber extracts (>50 %) gave positive signals, with melting temperatures that were not significantly different (<1 °C) from the true positive signal that is specific for *R. solanacearum*. Therefore, we concluded that the LAMP assay that targeted 16S rRNA is not suitable for detection of *R. solanacearum* in potato tubers.

As the specificity assay results were not optimal, the analytical sensitivity of the 16S rRNA LAMP assay was only tested on the standard curve of the spiked *R. solanacearum* DNA in potato extracts, and was estimated as 10^3^ to 10^4^ cells/mL (Table 3).

## 2. Optimized fliC LAMP assay

### *2.1 LAMP assay development*

For the LAMP assay that targeted *fli*C, an additional loop primer (5'ACATGACGGCTCCTATATTCC3') was designed according to the strategy described for LAMP primer design [22], with the aim to speed-up the previously described assay [23].

### *2.2 LAMP reactions*

LAMP reactions were performed as described in the Methods, using primers that have been published previously, with the addition of another loop primer and modified final primer concentrations, as described in Table S2.

Table S2. Primers used in the modified *fli*C LAMP assay.

| **Primer** | **5’-3’ primer sequence** | **Final primer conc.**  **(**µM**)** |
| --- | --- | --- |
| rsfliC_F3* | TTCAAGTTGCAGGTCACGT | 0.2 |
| rsfliC_B3* | AGGTTGTTTTCAACCTGGCC | 0.2 |
| rsfliC_FIP* | GAGATGTTGGTATTGAGGCTGAGCAAGCATTCACTCGGGCA | 1 |
| rsfliC_BIP* | GCCTGACCACGACCCTGAACAGGTACGAGTTCGCACCGT | 1 |
| rsfliC_loopF | ACATGACGGCTCCTATATTCC | 1 |
| rsfliC_loopB* | CGCAAACGCAAGGTATCCAGA | 1 |

* Primers previously published by Kubota *et al*., 2008 [23].

### *2.3 Analytical specificity*

The analytical specificity was determined on boiled suspensions of 10^8^ cells/mL, as determined by turbidity measurement (DEN-1B McFarland Densitometer, Biosan). Comparisons with real-time PCR were carried out as described in the Methods.

### *2.4 Results*

The previously published *fli*C LAMP assay was designed so that it makes use of only one loop primer [23]. The addition of a second loop primer especially designed for this study, and the use of an optimized primer concentration for the master mix described here, shortened the time needed for the *fli*C LAMP reaction by 5 min, to 10 min, as tested on nine *R. solanacearum* strains (data not shown). Based on the criteria described in the Methods (see main text), the running time was set to 40 min for the modified *fli*C LAMP assay.

The analytical specificity of the modified *fli*C LAMP assay was tested on the bacterial strains listed in Table S4. Three strains of *R. solanacearum* (NCPPB4161, NCPPB2088, NCPPB4153) showed no signals. Significantly, these three strains were isolated from potato and therefore are economically relevant. Furthermore, the potential cross-reacting strain of *R. pickettii* (NCPPB4165) generated a signal that could not be differentiated from true positives by the time of positivity or the T_m_ range for positive signals. The characteristic melting temperature for the modified *fli*C LAMP amplicon was experimentally determined at around 93.0 ±0.3 °C for phylotypes I, II and III, with one strain that had a significantly higher T_m_ of 93.9 °C, which was isolated from tomato in the USA. The majority of strains that belonged to phylotype IV showed a *fli*C LAMP amplicon melting temperature of around 93.5 ±0.2 °C (Figure 1B). Altogether, the range of T_m_ found with the modified *fli*C LAMP assay for the *R. solanacearum* species was between 92.2 °C and 93.9 °C, thus defining the criterion of LAMP signal acceptability (true positive)

The modified *fli*C assay also showed positive signals of amplification on three samples of DNA extracted from healthy potato extract (Table S5). However, the melting curve analysis of these positive signals showed significantly different melting temperatures (difference of 1 °C or more) compared to the melting curve defined for the true positive signals. Based on the defined criteria of positivity, these data were classified as negative, and did not cause problems in the results interpretation.

The analytical sensitivity of the modified real-time *fli*C LAMP assay was estimated as 10^5^ to 10^6^ cells/mL when tested on strains belonging to phylotypes I and III, 10^7^ cells/mL with phylotype IIA, 10^5^ cells/mL with phylotype IIB, and 10^8^ cells/mL with phylotype IV (Table S3). Next, the analytical sensitivity of the modified *fli*C LAMP assay in potato extracts was estimated to be 10^5^ cells/mL (Table 3), which is comparable to the previously reported sensitivity based on gel electrophoresis detection [23] and also to the analytical sensitivity obtained in water with a strain belonging to phylotype IIB.

Table S3. Analytical sensitivity of the modified *fli*C LAMP assay in different *R. solanacearum* phylotypes. Bacteria diluted in distilled water.

| **Concentration (cells/mL)** | **t_p_ (min)** | | | | |
| --- | --- | --- | --- | --- | --- |
|  | **GBBC 1172**  **(Phylotype I)** | **RUN 30**  **(Phylotype IIA)** | **GBBC 729 (Phylotype IIB)** | **LMG 2296 (Phylotype III)** | **RUN 71**  **(Phylotype IV)** |
| 10^8^ | 16.2 ±0.78 | 16.3 ±0.49 | 12.3 ±0.10 | 13.4 ±0.38 | 22.5 ±0.58 |
| 10^7^ | 19.3 ±0.62 | 24.7 ±10.19 | 13.2 ±0.29 | 16.7 ±0.44 | - |
| 10^6^ | 23.5 ±2.34 | - | 15.4 ±0.71 | 20.5 ±1.32 | - |
| 10^5^ | 32.1^a^ | - | 18.4 ±2.10 | 23.2^a^ | - |
| 10^4^ | - | - | - | - | - |
| 10^3^ | - | - | - | - | - |
| 10^2^ | - | - | - | - | - |
| T_m_ (°C) | 92.5 ±0.1 | 92.7 ±0.3 | 92.5 ±0.1 | 92.4 ±0.2 | 93.5 ±0.1 |

t_p_: time of positivity given in minutes (min).

“-“: negative result (absence of signal).

^a^ detected once out of three replicates.

Table S4. Bacterial strains tested with LAMP targeting 16S rRNA, *fli*C and *egl* compared to real-time PCR [19].

| **Strain, phylotype, collection** | **Origin** | **Host** | **Method** | | | | | | |
| --- | --- | --- | --- | --- | --- | --- | --- | --- | --- |
|  |  |  | **real-time PCR** | **LAMP assay** | | | | | |
|  |  |  | (10^6^ cells /mL) | **16S rRNA**  (10^6^ cells/mL) | | ***fli*C**  (10^8^ cells/mL) | | ***egl***  (10^8^ cells/mL) | |
|  |  |  | (C_q_) | **t_p_**  (min) | **T_m_**  (°C) | **t_p_**  (min) | **T_m_**  (°C) | **t_p_**  (min) | **T_m_**  (°C) |
| ***Ralstonia solanacearum* (phylotype not determined)** | | | | | | | | | |
| NCPPB3857^b^ | UK | *S. tuberosum* | 27.6 | 31.2 | 89.4 | 16.2 | 92.9 | 19.6 | 94.0 |
| NCPPB4154^b^ | Turkey | *S. tuberosum* | 27.6 | 35.4 | 89.3 | 16.0 | 93.1 | 17.6 | 93.9 |
| NCPPB4157^b^ | France | *S. tuberosum* | 26.7 | 33.3 | 89.4 | 15.3 | 93.0 | 17.3 | 93.7 |
| NCPPB4161^b^ | Germany | *S. tuberosum* | 26.8 | 35.4 | 89.8 | - | - | 19.6 | 93.6 |
| ***Ralstonia solanacearum* phylotype I** | | | | | | | | | |
| CFBP7058^c^ | Cameroon | *S. nigrum* | 28.6 | 33.1 | 88.4 | 25.8 | 92.8 | 17.3 | 94.4 |
| LMG17144^a^ | Fiji | *S. tuberosum* | 27.8 | 34.3 | 89.9 | 24.3 | 92.8 | 20.2 | 94.9 |
| GBBC1172^a^ | China | ND | 30.7 | 45.4 | 89.5 | 16.0 | 92.6 | 12.8 | 94.2 |
| NCPPB3996^a^ | Peru | *L. esculentum* | 26.9 | 35.5 | 89.4 | 27.1 | 93.2 | 18.8 | 94.8 |
| NCPPB4005^b^ | Philippines | *Zingiber* | 28.0 | 36.4 | 89.5 | 20.2 | 93.1 | 16.8 | 94.5 |
| RUN 47/CIP365^c^ | Philippines | *S. tuberosum* | 29.4 | 33.7 | 89.6 | 25.9 | 93.0 | 17.7 | 94.7 |
| RUN 54/CFBP6424^c^ | Guayana | *L. esculentum* | 28.5 | 34.1 | 89.8 | 26.1 | 92.9 | 17.6 | 94.6 |
| RUN 90^c^ | China | *Morus alba* | 28.6 | 36.9 | 89.4 | 26.2 | 92.8 | 16.5 | 94.5 |
| RUN 91/CIP277^c^ | China | *Morus alba* | 27.4 | 31.0 | 89.3 | 28.2 | 93.1 | 15.8 | 94.6 |
| RUN 159^c^ | Taiwan | *S. lycopersicum* | 29.6 | 36.2 | 89.5 | 39.9 | 92.7 | 17.6 | 94.7 |
| RUN 257^c^ | Taiwan | *S. lycopersicum* | 29.0 | 35.8 | 89.5 | 27.5 | 93.0 | 17.1 | 94.6 |
| RUN 258^c^ | Taiwan | *S. lycopersicum* | 28.5 | 36.1 | 89.3 | 25.9 | 93.0 | 16.6 | 94.6 |
| RUN 320^c^ | Madagascar | *Capsicum annum* | 28.1 | 33.5 | 89.6 | 26.4 | 93.2 | 18.0 | 94.8 |
| RUN 337^c^ | China | *Arachis hypogaea* | 27.9 | 32.8 | 89.3 | 23.8 | 93.0 | 18.6 | 94.8 |
| RUN 339^c^ | China | *Olea Europae* | 29.1 | 31.6 | 89.3 | 32.4 | 93.0 | 18.3 | 94.6 |
| RUN 343^c^ | China | *Morus alba* | 29.3 | 34.9 | 89.7 | 36.2 | 92.7 | 17.8 | 94.7 |
| RUN 471^c^ | Reunion | *Pelargonium* | 28.9 | 30.9 | 89.5 | 28.4 | 92.8 | 17.2 | 94.6 |
| RUN 597^c^ | Indonesia | *S. lycopersicum* | 28.1 | 28.9 | 89.2 | 26.1 | 92.9 | 17.6 | 94.9 |
| NCPPB3997^b^ | Australia | *S.tuberosum* | 27.6 | 29.0 | 89.3 | 23.8 | 93.0 | 15.9 | 94.6 |
| RUN 158^c^ | Australia | *Zingiber officinale* | 28.5 | 33.8 | 89.6 | 24.5 | 92.7 | 17.6 | 94.5 |
| ***Ralstonia solanacearum* phylotype IIA** | | | | | | | | | |
| RUN 288/CFBP6779^c^ | Martinique | *Canna indica* | 28.5 | 32.7 | 89.3 | 17.4 | 92.9 | 17.1 | 93.6 |
| AW 1^a^ | USA | *S.lycopersicum* | 27.4 | 37.4 | 89.1 | 9.5 | 92.5 | 12.1 | 93.2 |
| RUN 150/CFBP7032^c^ | Cameroon | *L. esculentum* | 28.6 | 35.3 | 89.6 | 20.0 | 93.3 | 18.2 | 93.8 |
| RUN 203/CFBP7054^c^ | Cameroon | *L. esculentum* | 37.3 | 40.2 | 89.0 | 21.4 | 92.9 | 20.6 | 94.4 |
| RUN 9^c^ | Hawaii | *Heliconia rostrata* | 28.3 | 33.8 | 89.7 | 19.1 | 93.0 | 16.1 | 93.6 |
| RUN 28/CFBP2958^c^ | Guadaloupe | *L. esculentum* | 28.8 | 32.0 | 89.9 | 19.0 | 93.3 | 18.2 | 94.0 |
| RUN 30/CFBP2972^c^ | Martinique | *S. tuberosum* | 28.8 | 34.2 | 89.7 | 16.2 | 92.9 | 14.6 | 93.8 |
| RUN 36/CFBP2957^c^ | Martinique | *L. esculentum* | 32.3 | 31.7 | 89.7 | 22.4 | 92.9 | 17.5 | 94.0 |
| RUN 43/CIP239^c^ | Brazil | *S. tuberosum* | 28.0 | 30.2 | 89.6 | 15.7 | 92.9 | 15.9 | 93.7 |
| RUN 45/CFBP3104^c^ | Peru | *S. tuberosum* | 30.3 | 32.8 | 89.7 | 18.3 | 92.8 | 17.1 | 93.8 |
| RUN 55/CFBP6431^c^ | Kenya | *S. tuberosum* | 29.0 | 31.0 | 89.3 | 16.9 | 93.0 | 17.5 | 93.8 |
| RUN 65/CFBP2047^c^ | USA | *Tomato* | 28.4 | 35.7 | 89.5 | 15.1 | 93.0 | 18.2 | 93.7 |
| RUN 301^c^ | Brazil | *Musa sp.* | 27.9 | 30.0 | 89.3 | 16.1 | 93.2 | 15.3 | 93.9 |
| RUN 448^c^ | Uruguay | *S. lycopersicum* | 28.4 | 32.9 | 89.5 | 27.2 | 93.0 | 17.6 | 93.9 |
| RUN 454/CFBP6438^c^ | Venezuela | *Musa sp.cv. plantain* | 27.7 | 33.2 | 89.4 | 20.3 | 93.2 | 16.0 | 93.8 |
| RUN 585^c^ | Grenada | *Musa sp.* | 28.3 | 27.7 | 89.2 | 19.4 | 93.0 | 16.3 | 93.8 |
| NCPPB 325^b^ | USA | *L. esculentum* | 27.8 | 27.7 | 89.0 | 18.2 | 93.9 | 19.9 | 93.9 |
| ***Ralstonia solanacearum* phylotype IIB** | | | | | | | | | |
| GBBC1112^a^ | Belgium | ND | 28.7 | 37.4 | 89.4 | 12.4 | 92.3 | 12.3 | 93.1 |
| GBBC729^a^ | Kenya | ND | 27.7 | 35.0 | 89.5 | 11.0 | 92.4 | 12.3 | 93.2 |
| NCPPB4160^b^ | Spain | *S. tuberosum* | 27.1 | 34.5 | 89.4 | 16.5 | 93.0 | 18.4 | 93.6 |
| NCPPB 3989^b^ | Brazil | *S. tuberosum* | 28.1 | 32.2 | 89.6 | 16.6 | 93.0 | 16.2 | 93.6 |
| RUN 1/CFBP6926^c^ | Netherlands | *S. tuberosum* | 27.9 | 27.6 | 89.7 | 17.1 | 93.0 | 18.6 | 93.7 |
| RUN 17/CFBP6783^c^ | Martinique | *Heliconia caribea* | 28.8 | 30.4 | 89.8 | 14.9 | 93.1 | 18.5 | 93.7 |
| RUN 40/CFBP6440^c^ | Peru | *S. tuberosum* | 28.3 | 32.8 | 89.7 | 17.5 | 92.8 | 17.4 | 93.7 |
| RUN 41/NCPPB 2088^c^ | Nigeria | *S. tuberosum* | 27.9 | 28.6 | 89.5 | - | - | 16.9 | 93.8 |
| RUN 74/CFBP6925^c^ | Philippines | *Musa x paradisiaca L. cv. Saba* | 28.8 | 36.1 | 89.5 | 16.2 | 92.9 | 16.1 | 93.8 |
| RUN 81/NCPPB3987^c^ | Brazil | *S. tuberosum* | 29.0 | 33.4 | 89.5 | 16.1 | 93.0 | 17.0 | 93.5 |
| RUN 147/CFBP7029^c^ | Cameroon | *S. lycopersicum* | 27.5 | 36.1 | 89.5 | 16.3 | 93.0 | 17.3 | 93.6 |
| RUN 262/CIP 70^c^ | Colombia | *Banana Plantain* | 28.2 | 34.7 | 89.4 | 16.1 | 93.0 | 17.1 | 93.6 |
| RUN 265/CIP418^c^ | Indonesia | *Arachis hypogaea* | 28.1 | 32.5 | 89.5 | 18.6 | 93.1 | 15.0 | 93.7 |
| RUN 297/CFBP7014^c^ | Trinidad | *Anthurium andreanum* | 27.7 | 33.1 | 89.4 | 13.3 | 93.0 | 15.9 | 93.8 |
| RUN 299^c^ | Brazil | *Pelargonium hortorum* | 30.8 | 36.7 | 89.3 | 15.4 | 92.8 | 16.5 | 93.8 |
| RUN 482/CIP240^c^ | Brazil | *S. tuberosum* | 28.3 | 32.4 | 89.4 | 20.9 | 93.0 | 15.5 | 93.7 |
| RUN 586^c^ | Peru | *Banan Plantain* | 28.0 | 34.9 | 89.4 | 14.8 | 92.9 | 16.7 | 93.8 |
| NCPPB1584^b^ | Cyprus | *S. tuberosum* | 29.1 | 32.2 | 89.5 | 15.5 | 93.0 | 18.9 | 93.9 |
| NCPPB2505^b^ | Sweden | *S. tuberosum* | 28.2 | 35.1 | 89.4 | 16.2 | 93.2 | 21.2 | 93.8 |
| NCPPB3985^b^ | Peru | *S. melongena* | 28.0 | 28.6 | 89.2 | 15.8 | 93.0 | 17.3 | 93.9 |
| NCPPB4028^b^ | Columbia | *L. esculentum* | 27.3 | 37.3 | 89.4 | 15.7 | 93.1 | 20.2 | 93.4 |
| NCPPB4153^b^ | Egypt | *S. tuberosum* | 28.1 | 34.3 | 89.9 | - | - | 20.03 | 93.71 |
| NCPPB4156^b^ | Netherlands | *S. tuberosum* | 27.3 | 34.5 | 89.5 | 15.6 | 93.0 | 17.4 | 93.9 |
| NCPPB4158^b^ | Portugal | *S. tuberosum* | 30.1 | 37.9 | 89.5 | 17.6 | 92.8 | 20.0 | 93.8 |
| ***Ralstonia solanacearum* phylotype III** | | | | | | | | | |
| RUN 166/CFBP7038^c^ | Cameroon | *S. nigrum* | 28.7 | 35.5 | 89.7 | 38.7 | 92.8 | 20.5 | 94.8 |
| CFBP3059^a^ | Burkina Faso | *S. melongena* | 27.5 | 35.7 | 89.5 | 14.3 | 92.6 | 17.1 | 93.5 |
| GBBC1222^a^ | Guinea | ND | 26.5 | 34.8 | 88.8 | 14.4 | 92.2 | 12.5 | 94.3 |
| LMG2296^a^ | Zimbabwe | *L. esculentum* | 27.5 | 35.8 | 88.6 | 14.3 | 92.2 | 12.3 | 94.2 |
| RUN 39/CFBP3059^c^ | Burkina Faso | *Eggplant* | 27.8 | 32.3 | 89.6 | 27.4 | 92.9 | 26.4 | 93.8 |
| RUN 56^c^ | Kenya | *S. tuberosum* | 28.4 | 37.6 | 89.6 | 33.5 | 92.5 | 16.5 | 94.7 |
| RUN 60/CFBP6434^c^ | Reunion | *Pelargonium asperum* | 28.2 | 35.7 | 89.4 | 25.9 | 93.0 | 18.9 | 94.5 |
| RUN 75/NCPPB332^c^ | Zimbabwe | *S. tuberosum* | 27.8 | 38.1 | 89.6 | 21.1 | 92.7 | 17.8 | 94.6 |
| RUN 76/NCPPB342^c^ | Zimbabwe | *Nicotiana tabacum* | 28.2 | 39.7 | 89.6 | 24.2 | 92.9 | 17.2 | 94.7 |
| RUN 133/CFBP6941^c^ | Cameroon | *S. lycopersicum* | 29.3 | 42.5 | 89.4 | 25.6 | 92.9 | 21.0 | 94.7 |
| RUN 145/CFBP6942^c^ | Cameroon | *Capsicum annuum* | 28.1 | 36.5 | 89.4 | 31.6 | 92.8 | 20.6 | 94.6 |
| RUN 362^c^ | Guinea | *S. tuberosum* | 28.1 | 33.3 | 89.4 | 23.9 | 92.8 | 19.4 | 94.7 |
| RUN 364^c^ | Guinea | *S. tuberosum* | 27.4 | 33.4 | 89.5 | 23.0 | 93.1 | 17.5 | 94.7 |
| RUN 369^c^ | Guinea | *S. tuberosum* | 27.1 | 34.4 | 89.5 | 24.4 | 92.9 | 17.8 | 94.8 |
| RUN479/NCPPB1018^c^ | Angola | *S. tuberosum* | 28.1 | 36.5 | 89.7 | 23.4 | 92.8 | 17.2 | 94.8 |
| ***Ralstonia solanacearum* phylotype IV** | | | | | | | | | |
| RUN 14^c^ | Australia | *S. lycopersicum* | NEG | 43.3 | 89.0 | 38.2 | 93.5 | - | - |
| RUN 71^c^ | Japan | *S. tuberosum* | 28.6 | 35.9 | 89.5 | 23.3 | 93.6 | 17.4 | 94.0 |
| RUN 83^c^ | Indonesia | *S. lycopersicum* | 28.5 | 36.6 | 89.5 | 23.1 | 93.4 | 17.8 | 93.9 |
| ***Ralstonia syzygii* phylotype IV** | | | | | | | | | |
| RUN 64^c^ | Indonesia | *Syzygumi aromaticum* | 27.0 | 32.8 | 89.6 | 25.2 | 93.2 | 15.8 | 94.5 |
| RUN 89^c^ | Indonesia | *Syzygumi aromaticum* | 29.2 | 38.5 | 89.5 | 22.4 | 93.6 | 27.1 | 94.0 |
| CFBP6447^a^ | Indonesia | *Syzygumi aromaticum* | 26.0 | 36.7 | 90.2 | 26.4 | 93.8 | - | - |
| **Blood disease bacterium phylotype IV** | | | | | | | | | |
| RUN 63^c^ | Indonesia | *Musa sp.* | 29.4 | 37.3 | 89.6 | 23.2 | 93.4 | - | - |
| NCPPB3726^b^ | Indonesia | *Musa hybrid banana* | 25.6 | 31.6 | 89.4 | 20.9 | 93.5 | - | - |
| ***Ralstonia pickettii*** | | | | | | | | | |
| SMT58^a^ | ND | ND | - | - | - | - | - | - | - |
| CFBP2459^a^ | USA | *Homo sapiens sapiens* | - | - | - | 33.7 | 92.3 | - | - |
| ***R. mannitolilytica*** | | | | | | | | | |
| CFBP6737^a^ | UK | *Contaminated autoclaved fluids* | 27.8 | - | - | - | - | - | - |
| **Other strains** | | | | | | | | | |
| *B. caryophilli* NCPPB353^b^ | USA | ND | - | - | - | - | - | - | - |
| *B. cepacia* NCPPB945^b^ | ND | ND | - | - | - | - | - | - | - |
| *B. plantarii* NCPPB3590^b^ | Japan | *Oryza sativa* | - | - | - | - | - | - | - |
| *B. andropogonis* NCPPB1127^b^ | Zimbabwe | *Bougainvillea sp.* | - | - | - | - | - | - | - |
| *B. glumae* NCPPB3708^b^ | Japan | *Oryza sativa* | - | - | - | - | - | - | - |
| *Paenibacillus polymyxa* NCPPB4162^b^ | France | *S. tuberosum* | - | - | - | - | - | - | - |
| *Pseudomonas marginalis pv. Marginalis* NCPPB4163^b^ | USA | *S. tuberosum* | - | - | - | - | - | - | - |
| *Burkholderia cepacia* NCPPB4164^b^ | USA | *Allium cepa* | - | - | - | - | - | - | - |
| *Enterobacter sp.* NCPPB4168^b^ | ND | ND | - | - | - | - | - | - | - |
| *Ochrobacterium anthropi* NCPPB4170^b^ | ND | ND | - | - | - | - | - | - | - |
| *Pectobacterium atrosepticum* NIB Z 5^d^ | ND | ND | ND | - | - | - | - | - | - |
| *Dickeya chrysanthemi* NCPPB 402^d^ | USA | *Chrysanthemum morifolium* | ND | - | - | - | - | - | - |
| *Pectobacterium spp.* NIB Z 83b^d^ | Slovenia | *S. tuberosum* | ND | - | - | - | - | - | - |
| *Pectobacterium spp.* NIB Z 85^d^ | Slovenia | *S. tuberosum* | ND | - | - | - | - | - | - |
| *Pectobacterium spp.* NIB Z 461^d^ | Slovenia | *S. tuberosum* | ND | - | - | - | - | - | - |
| *Dickeya solani* NIB Z 619^d^ | Slovenia | *S. tuberosum* | ND | - | - | - | - | - | - |
| *Pectobacterium atrosepticum* NIB Z 620^d^ | Slovenia | *S. tuberosum* | ND | - | - | - | - | - | - |
| *Dickeya solani* NIB Z 621^d^ | Slovenia | *S. tuberosum* | ND | - | - | - | - | - | - |
| *Dickeya solani* GBBC500^d^ | Belgium | *S. tuberosum* | ND | - | - | - | - | - | - |
| *Dickeya dianthicola* NIB Z 1824^d^ | ND | ND | ND | - | - | - | - | - | - |
| *Dickeya dieffenbachiae* NIB Z 1826^d^ | ND | ND | ND | - | - | - | - | - | - |
| *Dickeya chrysanthemi* NIB Z 1827^d^ | ND | ND | ND | - | - | - | - | - | - |
| *Clavibacter michiganensis subsp. sepedonicus* GSPB 1522^d^ | ND | ND | ND | - | - | - | - | - | - |

-, negative result (absence of signal).

ND, not determined.

^a^ Strain provided by ACW, Swiss Federal Research Station, Switzerland

^b^ Strain bought from National Collection of Plant Pathogenic Bacteria (NCPPB), FERA, UK

^c^ Strain provided by P. Prior, INRA/CIRAD UMR, Reunion, France

^d^ Collection of the National Institute of Biology, University of Ljubljana, Ljubljana, Slovenia

Table S5. Evaluation of LAMP performance on potato extracts (diagnostic samples).

| **Internal sample ID** | **Cultivar** | **Immunofluorescence *R.solanacearum* polyclonal antibodies^a^** | **real-time PCR**  **(Weller, 2000)** | **LAMP assay** | | | | | |
| --- | --- | --- | --- | --- | --- | --- | --- | --- | --- |
|  |  |  |  | **16S rRNA** | | ***fli*C** | | ***egl*** | |
|  |  |  |  | **t_p_**  (min) | **T_m_**  (°C) | **t_p_**  (min) | **T_m_**  (°C) | **t_p_**  (min) | **T_m_**  (°C) |
| D250/09 | Adora | NEG | - | - | - | - | - | 29.93 | 92.0 |
| D275/09 | Adora | SUSP | - | - | - | - | - | - | - |
| D2076/09 | Agria | SUSP | - | 41.2 | 88.6 | 33.8 | 89.9 | - | - |
| D797/10 | Aladin | NEG | - | 24.1 | 88.8 | - | - | - | - |
| D249/09 | Anuschka | SUSP | - | 27.5 | 87.9 | - | - | - | - |
| D792/10 | Arrow | NEG | - | - | - | - | - | - | - |
| D2071/09 | Arrow | NEG | - | 39.0 | 88.5 | - | - | - | - |
| D809/10 | Bella Rosa | NEG | - | 31.3 | 88.7 | - | - | - | - |
| D2047/09 | Bistra | NEG | - | - | - | - | - | - | - |
| D795/10 | Carlingford | SUSP | - | 30.6 | 88.5 | - | - | - | - |
| D800/10 | Carlingford | NEG | +(32.0 ±0.67) | 33.1 | 88.7 | - | - | - | - |
| D858/10 | Carlingford | NEG | +(29.6 ±0.16) | - | - | - | - | - | - |
| D2023/09 | Carrera | NEG | - | - | - | - | - | - | - |
| D819/10 | Cherie | NEG | - | - | - | - | - | - | - |
| D796/10 | Desiree | NEG | - | 34.3 | 88.7 | - | - | - | - |
| D811/10 | Desiree | NEG | - | - | - | - | - | - | - |
| D865/10 | Fiana | NEG | - | 28.0 | 88.7 | - | - | - | - |
| D810/10 | Frisia | NEG | - | 28.1 | 88.7 | - | - | - | - |
| D1068/09 | Frisia | NEG | - | 40.3 | 89.3 | - | - | - | - |
| D813/10 | Jelly | NEG | - | 27.5 | 88.5 | - | - | - | - |
| D814/10 | Jelly | NEG | - | - | - | - | - | - | - |
| D1066/09 | Jelly | NEG | - | - | - | - | - | - | - |
| D793/10 | Marabel | NEG | - | - | - | - | - | - | - |
| D815/10 | Marabel | NEG | +(39.1 ±NA) | 28.5 | 88.6 | 34.4 | 88.7 | - | - |
| D818/10 | Marabel | NEG | - | 48.1 | 88.7 | - | - | - | - |
| D862/10 | Marabel | NEG | - | 29.3 | 88.7 | - | - | - | - |
| D1065/09 | Marabel | NEG | - | 51.2 | 88.4 | 34.2 | 89.6 | - | - |
| D1067/09 | Marabel | NEG | - | 34.3 | 88.1 | - | - | - | - |
| D2024/09 | Marabel | NEG | - | - | - | - | - | - | - |
| D2051/09 | Mirna | NEG | - | 58.0 | 88.8 | - | - | - | - |
| D2049/09 | Pšata | NEG | - | - | - | - | - | - | - |
| D2072/09 | Sante | NEG | - | - | - | - | - | - | - |
| D2074/09 | Sante | NEG | +(28.7 ±0.13) | - | - | - | - | - | - |
| D2075/09 | Sante | NEG | - | - | - | - | - | - | - |
| D859/10 | Silvana | NEG | - | 35.5 | 88.9 | - | - | 20.5 | 90.6 |
| D798/10 | Sora | NEG | - | 27.6 | 88.6 | - | - | - | - |
| D808/10 | Sora | NEG | - | 34.5 | 88.5 | - | - | - | - |
| D2048/09 | Sora | NEG | - | - | - | - | - | - | - |
| D861/10 | Soraja | NEG | - | 36.0 | 90.1 | - | - | 28.9 | 92.2 |
| D799/10 | Unknown | NEG | - | - | - | - | - | - | - |
| D2050/09 | Unknown | NEG | - | 37.8 | 88.6 | - | - | - | - |
| D2052/09 | Unknown | NEG | - | 45.0 | 88.5 | - | - | 28.2 | 91.2 |
| D2070/09 | Unknown | NEG | - | - | - | - | - | - | - |
| D728/10 | Jelly | POS | + | NT | NT | NT | NT | 17.7 | 93.2 |
| D777/11 | Marabel | POS | + | NT | NT | NT | NT | 17.3 | 93.2 |
| D845/11 | Marabel | POS | + | NT | NT | NT | NT | 17.0 | 93.4 |
| D892/11 | Marabel | POS | + | NT | NT | NT | NT | 17.9 | 93.2 |
| D57/05 | Unknown | POS | + | NT | NT | NT | NT | 21.7 | 93.3 |
| D61/05 | Unknown | POS | + | NT | NT | NT | NT | 19.2 | 93.2 |
| D82/07 | Unknown | POS | + | NT | NT | NT | NT | 21.0 | 93.3 |

NEG, negative when tested with immunofluorescence.

SUSP, suspicious when tested with immunofluorescence, and confirmed negative using other methods.

NT, not tested.

^a^ Confirmed using all identification and confirmational tests (see Methods).

Table S6. Analytical sensitivity of the real-time PCR [19] for the different *R. solanacearum* phylotypes.

| **Concentration (cells/mL)** | **C_q_** | | | | |
| --- | --- | --- | --- | --- | --- |
|  | **GBBC 1172**  **(Phylotype I)** | **RUN 30**  **(Phylotype IIA)** | **GBBC 729 (Phylotype IIB)** | **LMG 2296 (Phylotype III)** | **RUN 71**  **(Phylotype IV)** |
| 10^8^ | 16.1 ±0.17 | 17.1 ±0.06 | 15.8 ±0.20 | 15.1 ±0.12 | 18.4 ±0.04 |
| 10^7^ | 21.6 ±0.03 | 23.4 ±0.13 | 21.2 ±0.14 | 20.3 ±0.04 | 24.5 ±0.24 |
| 10^6^ | 24.3 ±0.26 | 26.1 ±0.20 | 24.7 ±0.07 | 23.5 ±0.13 | 27.5 ±0.11 |
| 10^5^ | 27.5 ±0.65 | 30.3 ±0.48 | 28.0 ±0.11 | 27.4 ±0.04 | 31.2 ±0.04 |
| 10^4^ | 31.6 ±0.19 | 34.1 ±0.28 | 31.5 ±0.35 | 30.6 ±0.17 | 35.9 ±0.88 |
| 10^3^ | 34.9 ±0.76 | 34.9^a^ | 34.9 ±0.41 | 34.2 ±0.89 | - |
| 10^2^ | 36.4 ±1.21 | - | 37.8^a^ | 36.0^a^ | - |
| 10 | - | - | - | - | - |

Data are means ±standard deviation of triplicates.

Cq, quantification cycle.

“-“: negative result (absence of signal).

^a^ detected once out of three replicates

Table S7. Incubation times at 95 °C before the assays, and the following time to positivity and time taken for the *egl* LAMP assays with infected potato tuber.

| **Incubation time at 95 °C** (min) | **Time to positivity (t_p_)** (min:s) | **Total assay time** (min:s) |
| --- | --- | --- |
| 2 | 20:42 | 22:42 |
| 5 | 19:12 | 24:12 |
| 10 | 19:42 | 29:42 |
| 15 | 17:42 | 32:42 |
| 20 | 18:12 | 38:12 |
| 30 | 15:27 | 45:27 |

Table S8. Summary of the *egl* LAMP validation.

| **Factor** | **Results** | **Assay particulars** |
| --- | --- | --- |
| Analytical sensitivity | 20 copies of RS phylotype I DNA  2000 copies of RS phylotype IIA DNA  200 copies of RS phylotype IIB DNA  20 copies of RS phylotype III DNA  2000 copies of RS phylotype IV DNA | Sensitivity was assessed in triplicates on pure culture suspension on a single strain that represents each phylotype:   - Phylotype I (GBBC1172), limit of detection at 10^4^ cells/mL - Phylotype IIA (RUN 30), limit of detection at 10^6^ cells/mL - Phylotype IIB (GBBC729), limit of detection at 10^5^ cells/mL - Phylotype III (LMG 2296), limit of detection at 10^4^ cells/mL - Phylotype IV (RUN 71), limit of detection at 10^6^ cells/mL.   In addition, sensitivity was tested on two serial dilutions of DNA isolated from spiked tuber extracts: both showed limit of detection at 10^5^ cells/mL |
| Analytical specificity | 99% accurate (1 false negative, no false positives) | Tested on 88 strains of *R. solanacearum* species complex, 13 nontarget and potentially cross-reacting strains listed in the EU Council Directive 2009/29/EC and 13 other bacterial pathogens that can be present on *R. solanacearum* host plants. |
| Analytical selectivity | No cross-reactivity of different hosts/ cultivars/ tissues observed. | DNA from healthy plant extracts tested: potato (43 samples/21 cultivars), tomato (4), eggplant (3), pelargonium (6) and *S. dulcamara* (6). |
| Repeatability | 100% detection for dilutions with at least 10^4^ copies of Rs DNA or more. | Test was repeated 10 times on *R. solanacearum* GBBC1172 strain at 10^8^ cells/mL. Average t_p_ was 12.7 ±0.49 min, with T_m_ of 94.1 ±0.32 °C. Lower dilution (10^4^ cells/mL) was repeated three times, with times to positivity for strain BGGC1172 as follows: 23.2, 23.0 and 16.2 min. |
| Reproducibility | The test can be performed successfully with the three machines. | The test was performed on three different machines: SmartCycler (Cepheid, Sunnyvale, CA), Genie II (Optigene Ltd, Horsham, UK), and Roche Light Cycler 480 (Roche Applied Science). Results are comparable. However, due to different ways of obtaining T_m_ by GenieII machine, the range of T_m_ is ~1 °C lower compared to T_m_ for the SmartCycler and LightCycler 480. |
| Diagnostic specificity | All 50 samples tested with *egl* LAMP assay showed results in agreement with diagnostic status of the sample. | Seven real diagnostic samples that were previously confirmed to be *R. solanacearum* positive were positive using the *egl* LAMP assay. Forty-three samples of healthy potato extract (confirmed by real-time PCR and immunofluorescence) were negative using the *egl* LAMP assay. |
| Diagnostic sensitivity | Diagnostic sensitivity was estimated to be 10^5^ cells/mL | DNA isolated from three independently prepared serial dilutions of *R. solanacearum* in potato tuber extract were tested using the *egl* LAMP assay. The results were reproducible and diagnostic sensitivity was determined to be 10^5^ cells/mL. |
